# Isolated Osteomyelitis of the Thumb in the Setting of Colostomy Bag Change: A Case Report

**DOI:** 10.7759/cureus.46285

**Published:** 2023-09-30

**Authors:** Joseph Munoz-Soto, Alondra Gonzalez-Ocasio, Bashar Hammad, Payaum Fotovat-Ahmadi, John Zazaian

**Affiliations:** 1 Internal Medicine, University of Medicine and Health Sciences, West Bloomfield, USA; 2 Internal Medicine, McLaren Oakland Hospital, Pontiac, USA; 3 Sports Medicine, Oakland University William Beaumont School of Medicine, Royal Oak, USA

**Keywords:** hygiene, thumb, anaerobic gram-positive cocci, colostomy bag, osteomyelitis

## Abstract

Osteomyelitis is an inflammation of the bone tissue that results from an infection. Bacteria can reach the bone through the bloodstream. Predisposed individuals include immunocompromised patients, such as diabetics and HIV patients. Older age and trauma are common risk factors for osteomyelitis. We report a case of osteomyelitis where a male in his early seventies came to the Emergency Department with a right swollen finger without having any history of trauma or infection. With the patient's history, we could not find anything to explain the presenting symptoms' cause. While performing the physical exam, we noticed the patient had a colostomy bag and went in-depth on this topic. He says he cleans his colostomy bag daily with his right hand. After evaluating the patient, surgery was performed with an incision and drainage. Diagnosis was made via MRI images and wound culture results, which showed early developing osteomyelitis and anaerobic Gram-positive cocci. These bacteria are commonly found in the gastrointestinal tract. While discussing this case with infectious disease, we could not rule out that the cause of this patient's infection could be because of improper hygiene protocols while changing his colostomy bag. With this case report, we aim to raise awareness of the importance of having proper hygiene when cleaning colostomy bags, as this can alter our skin barrier and organisms can enter and establish bone infections.

## Introduction

Osteomyelitis is an inflammatory condition that affects the bones. It is secondary to infections such as bacteria, fungi, and mycobacteria. The most common cause of osteomyelitis is *Staphylococcus aureus* [1]. An X-ray must be performed to diagnose osteomyelitis, followed by an MRI- and CT-guided biopsy and blood cultures. Treatment includes antibiotics, which the culture will guide in the selection process based on sensitivity. Vancomycin and ceftriaxone are the empiric antibiotic regimens for covering Gram-positive and Gram-negative bacteria [2]. Certain diseases, such as cardiovascular and diabetes mellitus, tend to increase the incidence of osteomyelitis. Indications for surgery include the extent of disease, surgical hardware in the infected area, and patient comorbidities. If MRI shows extensive bone/soft tissue involvement or if antibiotics do not work, surgery is indicated. Surgeons may drain the infected area and proceed with a debridement of the diseased bone. Debridement involves removing necrotic material; tissue and bone samples may be obtained for culture and histology [3]. Osteomyelitis can be classified as acute or chronic and occurs at any age. In the acute setting, the duration tends to be less than two weeks and, in the chronic setting, more than six weeks [4-5].

Osteomyelitis in adults tends to have a chronic course and usually requires long-term treatment. It has a contiguous spread, which means the infection started from a nearby soft tissue infection, usually from a diabetic foot ulcer due to a lack of blood flow. In adults, osteomyelitis usually occurs in the vertebral bodies; thus, patients commonly present with back pain. Patients commonly present with swelling, erythema, increased temperature, and pain over the affected site. Appropriate management and continuous surveillance are needed to prevent infectious complications, such as abscesses, cellulitis, and sepsis [6].

## Case presentation

An African-American male in his early 70s, with a past medical history of diverticulosis with diverticulitis flare-ups and a past surgical history of partial colectomy with a colostomy bag placement, presented to the hospital with a painful, swollen right thumb. The pain started three days before presenting to the hospital and was constant. The pain was described as an eight out of 10 in severity and increased when the patient tried to move his thumb. The patient denied any history of trauma, injury, or lacerations to the hand or finger that could have compromised his skin. The patient also presented with an elevated blood pressure of 194/76 and a heart rate of 81. The patient had no antihypertensive medication before coming to the Emergency Department. The patient denied fever, chills, chest pain, or shortness of breath. The patient denied any history of tuberculosis (TB) or drug addiction or stayed at refugee camps or night shelters.

Physical examination

**Figure 1:**
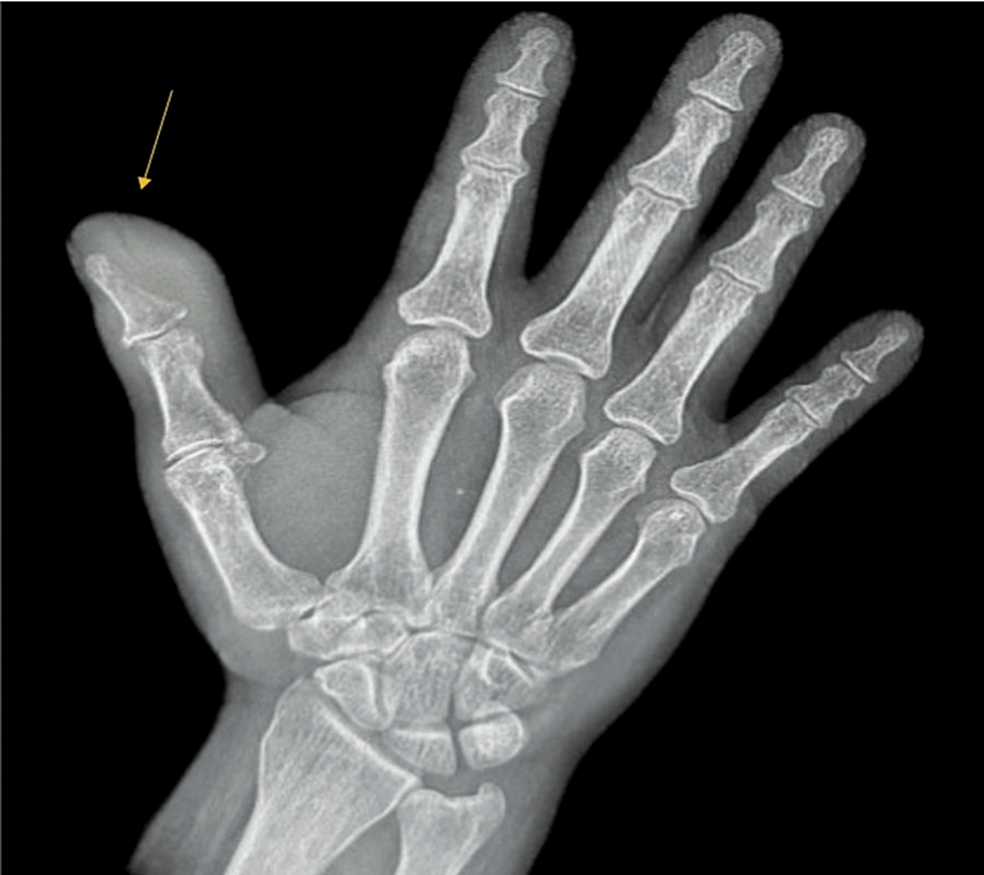
X-ray of the right hand showing soft tissue swelling involving the first digit.

The right thumb was erythematous, tender, and warm to the touch. No skin breakdown was noted. A radiologic examination of the right thumb was obtained, and it showed soft tissue swelling with cortical thinning concerning osteomyelitis. No fractures or foreign bodies were present (Figure 1).

**Figure 2:**
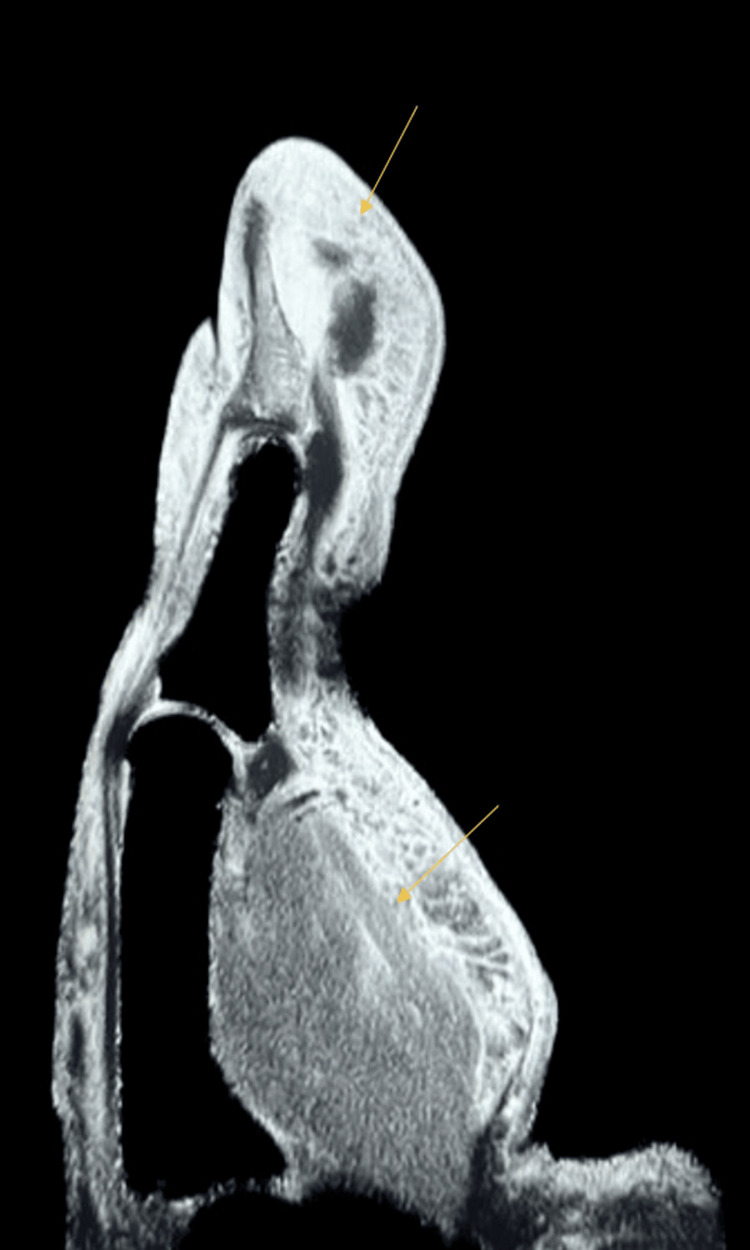
MRI of the first distal phalanx showing soft tissue swelling and edema.

Because of suspicion of osteomyelitis, the patient was empirically started on ceftriaxone and vancomycin. The Orthopedics team performed an incision and drainage of the thumb at the bedside for decompression. They drained approximately 2mL of yellowish purulent fluid. Wound and blood cultures were taken. Laboratory tests, such as complete blood count (CBC), comprehensive metabolic panel (CMP), c-reactive protein (CRP), and erythrocyte sedimentation rate (ESR), were ordered. CRP of 8.40 and an ESR of 102mm/hr were obtained. CBC showed WBC of 6.98 x 10^3/uL, Hgb of 13.8g/dL, and platelets of 203 x 10^3/uL, and CMP showed sodium of 138mmol/L, potassium of 4.3mmol/L, calcium of 9.2mg/dL, BUN of 21.2mg/dL, creatinine of 1.3mg/dL, glucose of 108mg/dL, ALT of 13U/L, and AST of 20U/L. The GeneXpert testing for TB was not detected. An MRI was also obtained, and it showed osteitis and/or early developing osteomyelitis of the first distal phalanx, partial thickness tear of the flexor tendon attachment site on the volar base of the first distal phalanx, and soft tissue swelling and edema (Figure 2).

After wound culture results were obtained, the Infectious Disease team decided to change the antibiotics to ertapenem and daptomycin when the anaerobic wound culture of the thumb returned positive for anaerobic Gram-positive cocci and blood culture for *Corynebacterium *species. Before the patient's discharge from the hospital, the patient was medically stable and was sent with an osteomyelitis treatment protocol of intravenous antibiotics via a peripherally inserted central catheter (PICC) line for six more weeks. The patient was also started on amlodipine 10mg daily to control blood pressure at home, with a reading of 128/73 at discharge. The patient was counseled on maintaining adequate hygiene on colostomy bag changes. He was walked through a standard step-by-step protocol for this process. The patient followed up two weeks after discharge on an outpatient basis with the involved orthopedic department after discharge from the hospital, and decreased localized pain was seen on review of systems, and significant clinical improvement was noted on the physical exam.

## Discussion

Osteomyelitis in the elderly has an approximate incidence of 21.8 per 100,000 individuals [7]. In addition, osteomyelitis mainly occurs in long bones and not in the bones of the fingers, as seen in this case [8]. Infections can travel through the bloodstream, reaching different parts of the body system and adhering to it. It can also occur via direct breach, which cannot be ruled out in this case. Colostomy bags can present various challenges, such as leaks, due to improper adhesion to the skin. Human feces contain digestive enzymes that can irritate the skin. As people age, the epidermal barrier decreases [9]. Prolonged exposure of feces to the skin can disrupt the epidermal barrier, causing pathogens to enter and adhere to local sites, leading to infection.

The patient's wound culture of the first distal phalanx was positive for anaerobic Gram-positive cocci. This group of bacteria can be found in several places throughout the body, including the gastrointestinal tract. The patient has a history of colostomy bag placement around five years ago; the patient's bag was changed regularly, and the patient had no past medical history or recent trauma. A relationship between the frequent bag changes and the presenting symptoms of the right thumb could be a high possibility. In addition, the patient was right-handed, making this his dominant hand used to manage the changes.

As the bag change occurs, the patient must follow a proper hygiene protocol, which the patient did not follow, and contamination can occur. As time passes and the appropriate measures are not taken, the continued contact of the feces and the patient's skin could have caused alteration in the skin barrier function, causing microorganisms to enter and establish in a local area. This is a case of acute osteomyelitis due to its timeline of presenting symptoms.

When a patient irrigates the stoma area with normal saline for cleaning, feces material could have come in contact with the skin. The safety protocol for colostomy bags must include gloves at all times, shaving areas around the stoma, and using powder to help the adhesive adhere firmly. By doing these steps, the colostomy bag should remain sealed and free from any feces leaking through it [10]. This is the first such case of osteomyelitis of the first distal phalanx secondary to poor hygiene with a colostomy bag change.

## Conclusions

Osteomyelitis is a disease that can have a good outcome, but it can also be life-threatening if left untreated or managed late in its course. This case report can bring awareness to not only the patients but also the health providers who aid in changing patients’ colostomy bags. It can be a risk factor for getting osteomyelitis if good hygiene is not followed; thus, using gloves and hand washing is crucial.
